# An Important Factor Affecting the UV Aging Resistance of PBO Fiber Foped with Nano-TiO_2_: The Number of Amorphous Regions

**DOI:** 10.3390/polym11050869

**Published:** 2019-05-13

**Authors:** Jiping Liu, Xiaobo Liu, Dong Wang, Hu Wang

**Affiliations:** 1 School of Materials Science and Engineering, Beijing Institute of Technology, Beijing 100081, China; liuxiaobo0425@126.com (X.L.); wd2263988@163.com (D.W.); wanghu123@bit.edu.cn (H.W.); 2 State Key Laboratory of Explosion Science and Technology, Beijing 100081, China

**Keywords:** titanium dioxide, poly(p-phenylene benzobisoxazole), ultraviolet aging, amorphous region

## Abstract

Modified nano-TiO_2_ was prepared by using triethanolamine and tetraisopropyl di (dioctylphosphate) titanate, respectively. Then the poly(p-phenylene benzobisoxazole) (PBO) fibers doped with different additions of modified nano-TiO_2_ particles were prepared by preparing PBO polymer solution and dry-jet wet spinning technique. Thermogravimetric and derivative thermogravimetry results showed that the addition of nano-TiO_2_ could improve the crystallinity and maximum thermal decomposition rate temperature of PBO fibers. Tensile strength results showed that nano-TiO_2_ addition did not affect the tensile properties of PBO fibers before ultraviolet (UV) aging began, and nano-TiO_2_ with addition values lower than 3% could improve the UV aging resistance performance of PBO fibers, while the aging resistance would be seriously reduced if values were over 5%. The size and quantity of the amorphous regions have a more important influence on the aging resistance of PBO fibers.

## 1. Introduction

Poly(p-phenylene benzobisoxazole) (PBO) fiber, a kind of rigid-rod isotropic crystal polymer, has excellent thermal stability, solvent resistance, remarkable tensile strength, and modulus [1,2,3]. PBO fiber known as a sort of synthesis material has become prominent in high strength applications, such as body armor, ropes and cables, and recreational equipment [4]. However, the ultraviolet (UV) aging resistance performance of PBO is poor. UV light irradiation was a frequently encountered factor that could induce photo-degradation of polymers in the outdoor environment [5,6]. PBO fiber was sensitive to UV light exposure, which could cause chemical, physical and mechanical properties changes.

Many studies have attempted to improve the aging resistance of PBO fiber [7,8,9,10]. Among them, introducing anti-ultraviolet agents received a great deal of attention [11,12,13]. As a strong UV absorber, nano-TiO_2_ has perfect versatility in optical, electrical, and photochemical properties [14,15,16,17], and can improve the resistance to UV aging of PBO fiber [18]. Many methods have been explored to prepare the nano-TiO_2_/PBO nanocomposites, such as sol–gel blending technique [19], in situ polymerization process [20,21] and solution blending [22]. However, nano-TiO_2_ has extremely large surface energy and is not easily dispersed in fiber matrix [23]. Meanwhile, the compatibility of PBO fibers with titanium dioxide particles is poor, and cracks and defects are easily produced at the interface between them. Therefore, achieving the compatibility between nano-TiO_2_ and PBO matrix and achieving uniform dispersion of nano-TiO_2_ in PBO matrix are the key problems.

To solve this problem, we used triethanolamine (TEA) and tetraisopropyl di (dioctylphosphate) titanate (TDT) to prepare modified nano-TiO_2_ via surface modification which can help to form chemical and physical interactions between two incompatible phases [12,24,25,26]. Then the modified PBO fiber with different TiO_2_ contents was synthesized using typical PBO polymerization conditions.

The characterization of modified nano-TiO_2_ and its effect on the properties of PBO fiber were determined in this study. Thermogravimetric (TG) and derivative thermogravimetry (DTG) were used to determine the thermal properties of modified nano-TiO_2_ and modified PBO fiber. The element contents of modified nano-TiO_2_ samples were determined by X-ray fluorescence (XRF). Furthermore, the microstructure of nanoparticles and fiber were evaluated via scanning electron microscopy (SEM) characterization of the surface morphology. In addition, Fourier transform infrared (FTIR) spectroscopy was used to further investigate the compositional changes of nano-TiO_2_ and PBO fiber. In addition, a schematic diagram of the influence mechanism of the size and quantity of crystal region on the aging resistance performance of PBO fiber was provided.

## 2. Experimental Procedure

### 2.1. Preparation of Modified Nano-TiO_2_

**Figure 1:**
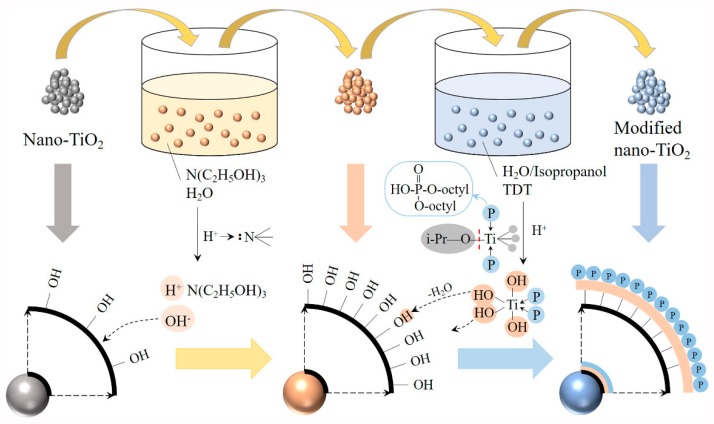
Procedure of preparing surface-modified nano-TiO_2_ particles.

The modified nano-TiO_2_ was prepared by surface modifying nano-TiO_2_ (99.9% purity, average particle size: 50 nm, mainly composed of rutile phase, Sinopharm Chemical Reagent Beijing Co., Ltd., Beijing, China) with TEA (99.7% purity, Sinopharm Chemical Reagent Beijing Co., Ltd., Beijing, China) and TDT (99.7% purity, Sinopharm Chemical Reagent Beijing Co., Ltd., Beijing, China), respectively. A certain number of nano-TiO_2_ particles in 65% TEA aqueous solution was first treated in an ultrasonic bath (frequency of 40 kHz, power 300W, GT SONIC, Guangdong, China) for 30 min, and then stirred for 2 h with reflux at 90 °C. Then the treated nano-TiO_2_ particles were filtered and dried at 100 °C for 6 h in a vacuum system. Subsequently, the treated nano-TiO_2_ particles were modified with TDT at 100 °C and on the pH value of 3.6 for 2 h according to the weight ratio of nano-TiO_2_, TDT, water, and isopropanol to 4:2:95:3. The modified nano-TiO_2_ particles were filtered and washed with distilled water three times and washed with ethanol one time and dried at 100 °C for 6 h in a vacuum system to remove the solvent. The procedure of preparing surface-modified nano-TiO_2_ is shown in Figure 1.

### 2.2. Preparation of PBO Fibers

The PBO fibers doped with different additions of modified nano-TiO_2_ particles were prepared as follows.

**Figure 2:**
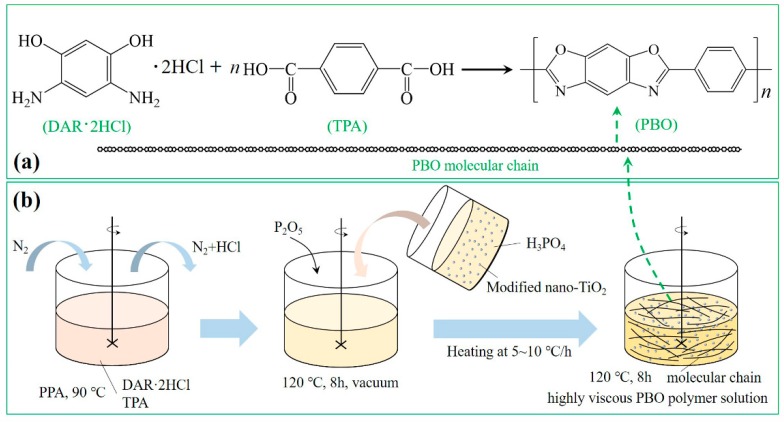
Scheme of PBO preparation (**a**), and procedure of preparing poly(p-phenylene benzobisoxazole) (PBO) polymer solution (**b**).

The PBO polymer solution was prepared by using the polycondensation of 4,6-diaminoresorcinol dihydrochloride (DAR·2HCl, 99.9% purity) and terephthalic acid (TPA, 99.9% purity, dried before use) doped with different additions (0%, 1%, 3%, 5%) of modified nano-TiO_2_ particles [27,28]. The poly (phosphoric acid) (PPA, with a phosphorus pentoxide content of 70.7 wt %) was loaded into a glass vessel equipped with a mechanical stirrer and nitrogen inlet/outlet. Then DAR·2HCl and TPA were placed in PPA and mixed together with PPA at 90 °C under a nitrogen (N_2_) atmosphere until complete removal of hydrochloride (HCl). The mole ratio of DAR·2HCl and TPA was 1:1. Modified nano-TiO_2_ particles which were placed in phosphoric acid (H_3_PO_4_) and treated with ultrasonic bath (40 kHz) for 30 min were added to the glass vessel. Phosphorus pentoxide (P_2_O_5_) was then added to the glass vessel to ensure the P_2_O_5_ concentration up to 85% and result in a final polymer concentration of 14%. The polymerizing mixture was first stirred under vacuum at 120 °C for 8 h and then heated to 180 °C stepwise at 5~10 °C per hour and kept at 180 °C for another 8 h with constant stirring. Then a highly viscous PBO polymer solution doped with nano-TiO_2_ particles was prepared (see Figure 2b). By changing the amount of modified nano-TiO_2_, we prepared four kinds of fibers with modified nano-TiO_2_ content of 0%, 1%, 3%, 5%, respectively. The scheme of PBO preparation was shown in Figure 2a and the procedure of preparing a PBO polymer solution was shown in Figure 2b.

**Figure 3:**
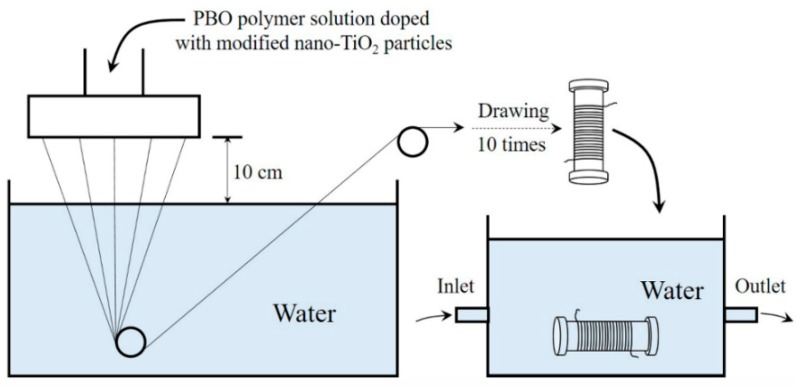
The procedure of dry-jet wet spinning of PBO fibers.

The obtained highly viscous PBO polymer solutions in PPA were then spun into PBO fibers using dry-jet wet spinning. The fiber exited into a 10 cm long air gap before entering a distilled water coagulation bath maintained at room temperature. Draw ratios as high as 10 were achieved. Fibers were wound on a plastic spool, washed in running water for a week, and subsequently dried overnight in a vacuum at 80 °C. The procedure of dry-jet wet spinning of PBO fibers is shown in Figure 3.

### 2.3. Characterizations and Measurements

The thermal decomposition performance of samples was determined via TG/DTG (Mettler Toledo, with a heating rate of 10 °C per minute, nitrogen atmosphere). FTIR (Bruker tensor27, Karlsruhe, Germany) was employed to characterize the chemical structure changes of modified nano-TiO_2_ particles and PBO fibers. Furthermore, the surface morphology of nano-TiO_2_ particles and PBO fibers were evaluated by using SEM (Hitachi S-4800, Tokyo, Japan). In addition, the UV exposure test of PBO fiber samples was carried out on a xenon lamp weatherometer (1500 W Xenon lamp, 380 nm UV rays, 250~765 W/m^2^). Tensile testing of fibers which were mounted on cardboard tabs was performed on the universal tensile tester (model WD-1) using two centimeter gage length at a strain rate of 2% per minute.

## 3. Results and Discussion

### 3.1. Characterizations of Modified Nano-TiO_2_

As Figure 1 shows, TEA causes the increase of hydroxyl ion (OH^−^) in the solution because of the existence of the lone pair electrons in the N atom. Owing to the high molecular polarity and high surface energy, OH^−^ ions were adsorbed on the surface of nano-TiO_2_ particles to form Ti(OH)_n_. The Ti–O bond of TDT would be hydrolyzed to the hydroxyl group (–OH) in an acid solution. The newly formed hydroxyl groups then reacted with the hydroxyl group on the surface of the TiO_2_ to form a chemical connection. Therefore, as Figure 1 shows, the surface modifier of modified nano-TiO_2_ was mainly from TDT.

**Figure 4:**
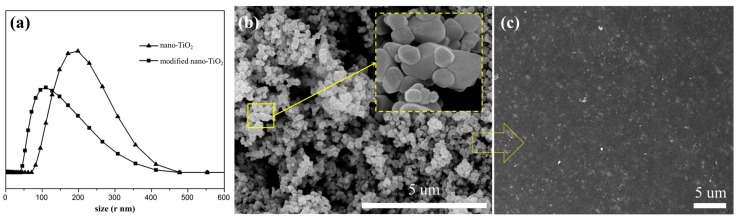
Particle size distribution of nano-TiO_2_ before and after modification (**a**), SEM images of modified nano-TiO_2_ (**b**), and the surface of PBO fiber containing modified nano-TiO_2_ particles (**c**).

The particle size distribution of nano-TiO_2_ before and after modification shown in Figure 4a. The surface micromorphology of the modified nano-TiO_2_ was evaluated via SEM shown in Figure 4b. As Figure 4b shows, the nano-TiO_2_ particles are roughly pebble-shaped with about 50 nm in average diameter. There were many evenly distributed spots on the surface of the nano-TiO_2_ particles, and they were caused by surface modification.

**Figure 5:**
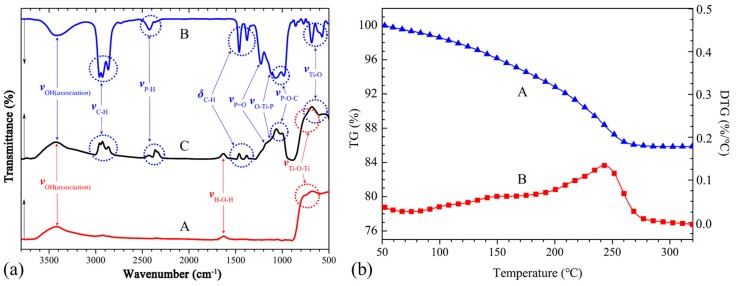
(**a**): FTIR spectra of pure nano-TiO_2_ (A), tetraisopropyl di (dioctylphosphate) titanate (TDT) (B), and modified nano-TiO_2_ (C), (**b**): TG (A) and derivative thermogravimetry (DTG) (B) of modified nano-TiO_2_ under nitrogen with a heating rate of 10 °C per minute.

The surface modification of nano-TiO_2_ was verified via the FTIR absorption spectra shown in Figure 5a. These spectra were used to determine the compositional changes of nano-TiO_2_ before and after modification. A curve in Figure 5a is the FTIR spectra of pure nano-TiO_2_. As A curve shows, the peak at ~779 cm^−1^ is the typical vibration of Ti–O–Ti. The broad peak at ~3420 cm^−1^ is the typical O–H stretching vibration peak corresponding to the associated hydroxyl group of Ti(OH)_n_. Similarly, the peak at ~1628 cm^−1^ is the typical H–O–H stretching vibration peak of the adsorbed water at the surface of nano-TiO_2_ particles. After the modification of TDT, the FTIR characteristics of TDT would be reflected in the spectrum of the modified nano-TiO_2_ (see B and C curve in Figure 5a). The peaks occurring at ~2960, 2927, and 2865 cm^−1^ correspond to the stretching vibration of C–H. The doublet peaks at ~1461 and 1378 cm^−1^ correspond to the bending vibration of C–H. Moreover, the peaks at ~2400, ~1229, ~1062, ~999, and ~700 cm^−1^ are typical of TDT. The FTIR results reveal that the TDT has been successfully chemically bonded to the surface of nano-TiO_2_ particles. TG and DTG were employed to determine the thermal performance and the content of modifier on the surface of the modified nano-TiO_2_ particle [29], and the results were shown in Figure 5b. As can be seen from Figure 5b, the modified nano-TiO_2_ shows continuous weight loss until about 260 °C. This can be attributed to physically adsorbed water volatilization and the thermal decomposition of modifier chains. Therefore, based on the result, the surface modifier content of nano-TiO_2_ particles was no more than about 14 wt %.

### 3.2. Effect of Modified Nano-TiO_2_ on UV Aging Resistance of PBO Fiber

To investigate the effect of modified nano-TiO_2_ on UV aging resistance of PBO fiber, the PBO fiber containing 0%, 1%, 3%, and 5% modified nano-TiO_2_ content were prepared, and they were named PBO-0-TiO_2_, PBO-1-TiO_2_, PBO-3-TiO_2_, PBO-5-TiO_2_, respectively.

In order to investigate the relationship between the nano-TiO_2_ content and the polymer molecular weight, the intrinsic viscosity [η] values of PBO polymer solutions were determined via Ubbelohde viscometer in Methanesulfonic acid (MSA) at 30 ± 0.2 °C. The [η] values of polymer solutions of PBO-0-TiO_2_, PBO-1-TiO_2_, PBO-3-TiO_2_, and PBO-5-TiO_2_ were 6.0, 6.0, 5.0, and 4.0 dL/g, respectively. According to the correlation of [η] and the weight-average molecular weight (*M*_w_) of PBO [25], *M*_w_ values of PBO-0-TiO_2_, PBO-1-TiO_2_, PBO-3-TiO_2_, and PBO-5-TiO_2_ were calculated as 2.5 × 10^4^, 2.5 × 10^4^, 2.2 × 10^4^, and 1.8 × 10^4^ g/mol, respectively. This result suggested that the molecular weight of PBO-1-TiO_2_ was the same as PBO-0-TiO_2_, and as the nano-TiO_2_ content increasing, the molecular weight of PBO polymer solution decreased gradually. This is because the incorporation of TiO_2_ reduced the chance of PBO chain combination, which resulted in the decrease of molecular weight of PBO.

**Figure 6:**
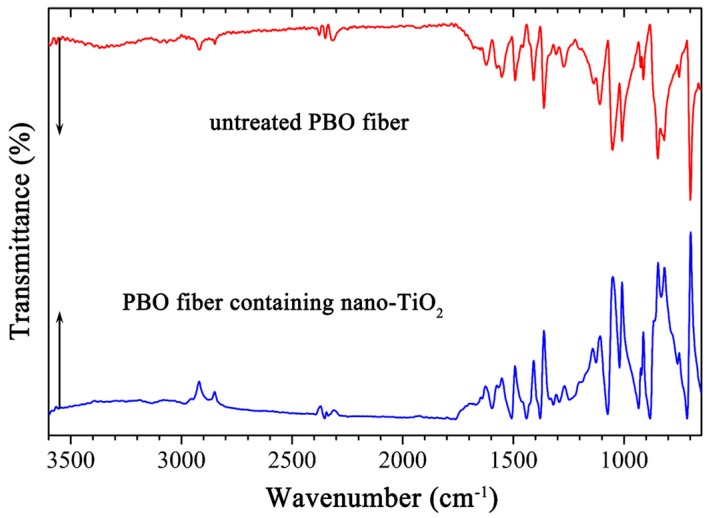
FTIR spectra of untreated PBO fiber and PBO fiber containing nano-TiO_2._

In order to investigate the effect of nano-TiO_2_ on the morphology and composition of PBO, the surface microstructure and the chemical structure changes of PBO fiber containing 5% nano-TiO_2_ were characterized via SEM shown in Figure 4b and via FTIR shown in Figure 6, respectively. As Figure 4b showed, the nano-TiO_2_ embedded in the PBO matrix and even individual particles can be observed, nano-TiO_2_ was homogeneously distributed in the PBO matrix without forming significant aggregation. As Figure 6 showed, there was no difference in the FTIR spectra between untreated PBO fiber and PBO fiber containing modified nano-TiO_2_. As the TiO_2_ added, the absorption bands of modified PBO fiber were also the same as untreated PBO fiber. It means that the structure of PBO fiber was not obviously changed, and TiO_2_ was physically dispersed in the PBO matrix and did not form a chemical connection.

**Figure 7:**
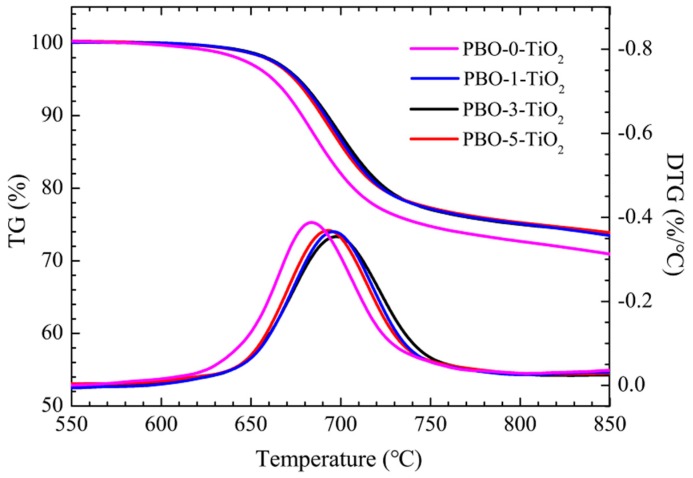
Thermogravimetric (TG) and DTG of PBO fibers under N_2_ with a heating rate of 10 °C per minutes.

TG and DTG were employed to determine the thermal performance and the effect of modified nano-TiO_2_ on PBO fibers of PBO-0-TiO_2_, PBO-1-TiO_2_, PBO-3-TiO_2_, and PBO-5-TiO_2_, respectively. The TG and DTG results were shown in Figure 7. As Figure 7 showed, all the fibers exhibited outstanding thermal stability with no appreciable weight loss below 600 °C. The initial thermal decomposition temperature of pure PBO fiber was lower than that of PBO fibers containing modified nano-TiO_2_. The maximum thermal decomposition rate temperature of PBO-0-TiO_2_, PBO-1-TiO_2_, PBO-3-TiO_2_, and PBO-5-TiO_2_, was 684.2, 693.3, 696.2, and 698.1 °C, respectively. The TG and DTG results showed that nano-TiO_2_ can help to improve the heat resistance of PBO fiber. As mentioned above, the molecular weight of PBO-0-TiO_2_ was higher than that of the other three PBO fibers, and the molecular weight and degree of crystallinity were the main factor affecting the thermal decomposition properties of polymers. Therefore, the addition of nano-TiO_2_ can improve the crystallinity of the PBO fibers.

**Figure 8:**
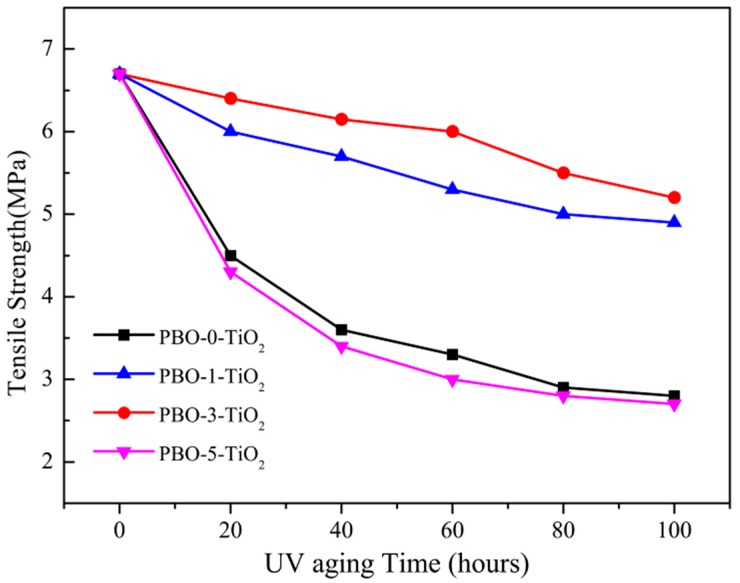
Tensile strength of the PBO fibers after UV aging for different times.

**Figure 9:**
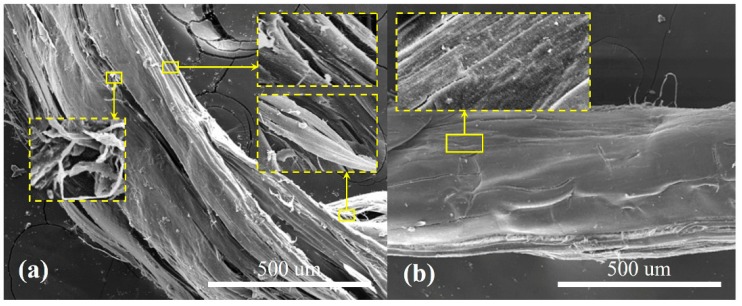
SEM images of PBO-0-TiO_2_ fibers (**a**) and PBO-3-TiO_2_ fibers (**b**) after exposure to UV-light for 100 h.

In order to investigate the effect of nano-TiO_2_ on UV aging resistance properties of PBO fiber, the tensile strength and surface morphology of PBO fibers after UV radiation aging were characterized via universal tensile tester and SEM shown in Figure 8 and Figure 9, respectively.

Figure 8 showed the relationship between the tensile strength of different PBO fibers and the exposure time of UV radiation aging. It was obvious that the nano-TiO_2_ had a good UV protection effect on PBO fiber, and PBO-3-TiO_2_ had the best UV aging resistance properties. The comparison of fiber morphology after aging between PBO-3-TiO_2_ and PBO-0-TiO_2_ was shown in Figure 9. As Figure 9 shows, after UV aging, the surface of PBO-0-TiO_2_ fibers became rough and the cracks were obvious (see Figure 9a), while the PBO-3-TiO_2_ fiber mainly maintained the original morphology with only slight cracks (see Figure 9b). This indicated that the nano-TiO_2_ improved the UV aging resistance properties of PBO fiber.

Furthermore, Figure 8 results demonstrate that the tensile strengths of four PBO fibers were the same before the beginning of UV aging, and were decreased with the increase of UV aging time. Although the content of nano-TiO_2_ in PBO-5-TiO_2_ is higher, it is remarkable that the anti-aging properties of PBO-5-TiO_2_ are worse than PBO-5-TiO_2_, even worse than PBO-0-TiO_2_. This indicates that the different additions of nano-TiO_2_ have no influence on the tensile strengths of four PBO fibers without UV aging, but has a great influence on the UV anti-aging properties of PBO fiber. This result was discussed in two aspects:

Firstly, this was because the four PBO fibers had different molecular weights, chain orientations, and crystallinities. The PBO-0-TiO_2_ has highest molecular weight, best chain orientation and least crystallinity. With the addition of nano-TiO_2_, the molecular weight of PBO became smaller, the molecular chain orientation became worse, but the crystallinity became higher. Therefore, with the combined effect of three factors, there was no obvious change in the strength of PBO fibers before the beginning of UV aging.

**Figure 10:**
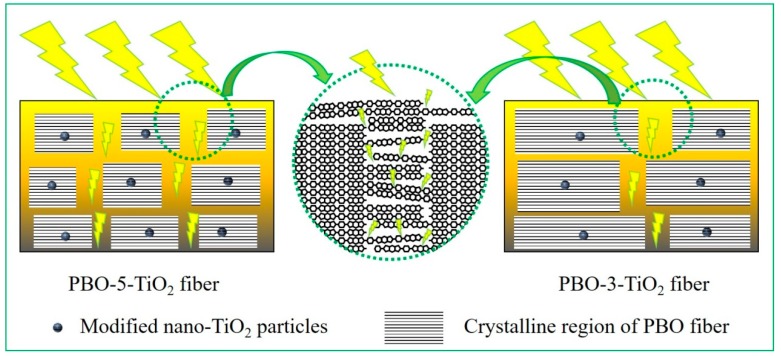
Schematic diagram of the influence of crystal region distribution on UV aging resistance.

Secondly, it is worth noting that nano-TiO_2_ could induce crystallization as a heterogeneous nucleation agent. More additive amount of nano-TiO_2_ means more nuclei in the PBO fiber, and each single crystal region is smaller. Therefore, although PBO-3-TiO_2_ and PBO-5-TiO_2_ had similar crystallinity, the quantity and size of their crystal regions were different. This further means that the quantity and size of the amorphous region were different, which were the weak areas of ultraviolet aging and could be seen as the PBO fiber in a specific UV irradiation environment (see Figure 10). Therefore, although PBO-5-TiO_2_ and PBO-3-TiO_2_ have similar initial strength, after a period of UV aging, PBO-5-TiO_2_ showed the worst UV aging resistance.

## 4. Conclusions

In this research, modified nano-TiO_2_ was prepared by using TEA and TDT, respectively. Then the PBO fibers doped with different content modified nano-TiO_2_ particles were prepared by preparing PBO polymer solution and dry-jet wet spinning technique. Then we studied the effect of nano-TiO_2_ content on the properties of PBO fiber. The main conclusions of the research are as follows:

(1) TDT has a good modification effect on nano-TiO_2_ particles, and the modified nano-TiO_2_ particles were dispersed uniformly in the PBO matrix without forming new chemical bonds.

(2) Nano-TiO_2_ with the addition values less than 3% could improve the anti UV aging properties of PBO fibers, while the aging resistance properties of PBO will be seriously reduced if the content of nano-TiO_2_ is over 5%. However, nano-TiO_2_ with the addition of no more than 5% does not affect the tensile properties of PBO fibers before the beginning of UV aging.

(3) Nano-TiO_2_ could reduce the molecular weight and the orientation degree of PBO molecular structure and could increase the crystallinity of PBO fiber via inducing crystallization. With the increase of nano-TiO_2_ content (over 5% content), there will be more nucleation agent and more small crystalline regions, and this will lead to more small amorphous regions and defects which are the weak regions of UV aging.

(4) Crystallinity, molecular weight and molecular chain orientation play an important role in the strength of PBO fibers before the beginning of UV aging, but for the PBO fibers irradiated with ultraviolet, the size and quantity of crystalline regions may have a more important influence.
